# Lactation Stage-Dependency of the Sow Milk Microbiota

**DOI:** 10.3389/fmicb.2018.00945

**Published:** 2018-05-11

**Authors:** Wei Chen, Jiandui Mi, Ning Lv, Jinming Gao, Jian Cheng, Ruiting Wu, Jingyun Ma, Tian Lan, Xindi Liao

**Affiliations:** ^1^College of Animal Science, National Engineering Research Center for Breeding Swine Industry, South China Agricultural University, Guangzhou, China; ^2^Guangdong Provincial Key Lab of Agro-Animal Genomics and Molecular Breeding, Guangzhou, China; ^3^Ministry of Agriculture Key Laboratory of Tropical Agricultural Environment, South China Agricultural University, Guangzhou, China; ^4^Key Laboratory of Animal Health Aquaculture and Environmental Control, Guangzhou, China

**Keywords:** milk, sow, lactation stage, microbiota, diversity

## Abstract

Breast milk is essential for the initial development of neonatal animals, as it provides not only essential nutrients and a broad range of bioactive compounds but also commensal bacteria. The milk microbiota contributes to the “initial” intestinal microbiota of infants and also plays a crucial role in modulating and influencing neonatal health. However, the milk microbiota of sows has yet to be systematically investigated. The goal of the present study was to characterize variations in bacterial diversity and composition in sow milk over the duration of lactation using a high-throughput sequencing approach. Milk samples (*n* = 160) were collected from 20 healthy sows at eight different time points, and microbial profiles were analyzed by 16S ribosomal RNA (rRNA) sequencing using the Illumina MiSeq platform. The composition and diversity of the milk microbiota changed significantly in colostrum but was relatively stable in transitional and mature milk. *Firmicutes* and *Proteobacteria* were the most dominant phyla in sow milk. The relative abundances of the two most dominant bacterial genera, *Corynebacterium* and *Streptococcus*, were significantly higher in colostrum than in transitional milk and mature milk samples, and the other four most dominant bacterial taxa (*Lactobacillus*, two unclassified genera in the families *Ruminococcaceae* and *Lachnospiraceae*, and an unclassified genus in the order *Clostridiales*) demonstrated higher relative abundances in transitional and mature milk than in colostrum. Membrane transport, amino acid metabolism and carbohydrate metabolism were the most abundant functional categories in sow milk communities. Microbial network analysis based on the predominant genera revealed that the abundance of *Helcococcus* was negatively correlated with the abundances of most other genera in sow milk. Our results are the first to systematically indicate that the sow milk microbiota is a dynamic ecosystem in which changes mainly occur in the colostrum and remain generally stable throughout lactation.

## Introduction

Breast milk is the most important postpartum element during the initial development of neonates because it provides optimal nutrition, bioactive components, and host defense proteins to suit all needs of the developing neonate in an age-adapted manner (Walker, 2010; Ballard and Morrow, 2013). More recently, breast milk has been recognized as an important source of commensal bacteria that are able to act as pioneer bacteria during the critical stage of initial neonatal gut colonization (Fernandez et al., 2013; Li et al., 2017). Many of these commensal bacteria in milk play active roles in reducing the incidence and severity of infections (Martín et al., 2005; Olivares et al., 2006a), modulating early immune system development, reducing the risk of immune inflammatory or metabolic diseases (Olivares et al., 2006b; Diaz-Ropero et al., 2007), and determining metabolism in the infant (Maldonado et al., 2010; Gil-Campos et al., 2012).

An infant consumes approximately 800 mL of milk/day, ingesting between 1 × 10^5^ and 1 × 10^7^ bacteria daily, determined by culture-dependent methods (Heikkila and Saris, 2003). Recently, culture-independent molecular techniques, particularly those based on 16S rRNA genes, have also been used to characterize complementary biodiversity in the human (Hunt et al., 2011; Ward et al., 2013) and ruminant milk microbiomes (McInnis et al., 2015). *Staphylococcus*, S*treptococcus*, and *Pseudomonas* are the “dominant genera” in human milk (Jimenez et al., 2015; Fitzstevens et al., 2017). Using pyrosequencing to investigate the DNA encoding the V1–V3 hypervariable regions of bacterial 16S rRNA, Cabrera-Rubio et al. (2012) found that *Leuconostoc, Weissella, Lactococcus*, and *Staphylococcus* were predominant in mature milk produced by women living in Finland, while *Streptococcus* was more relatively abundant in colostrum. Boix-Amoros et al. (2016) used similar methods and found that the most common genera in the colostrum of Spanish mothers were *Staphylococcus* and *Acinetobacter*, while *Pseudomonas* and *Streptococcus* were most common in transitional milk and *Acinetobacter* was most common in mature milk samples.

Pork was the first meat consumed worldwide, either as fresh meat or various processed products. In addition, due to the high degree of similarity between the anatomy and nutritional physiology of humans and pigs, pigs have been extensively used as an outstanding model to explore the factors influencing gastrointestinal physiology and immune and brain development in humans (Szyndler-Nedza et al., 2013). One of the factors influencing the profitability of pork production is the normal development of piglets, which is determined principally by the amount and quality of milk produced by sows because milk is the main food for piglets during the first 3 weeks of their lives. Thus, many studies have been performed to determine the effects of various factors on nutritional and protective compounds in sow milk (Velayudhan and Nyachoti, 2017; Wang et al., 2017). The intestinal microbiota is a critical factor for host health. Accumulating evidence suggests that the development of the gut microbiota during early life sets the stage for the adult microbiome and has long-term impacts on the health of the host (Turnbaugh et al., 2009; Han, 2015). However, no studies until this work have systematically investigated the milk microbiota of sow using next-generation sequencing technologies.

The objective of this study was to characterize variations in the bacterial diversity and composition of sow milk throughout lactation using next-generation sequencing technologies. In addition, the potential correlation between nutritional components and microbiota community was evaluated.

## Materials and Methods

### Ethics Statement

The experimental design and procedures followed the institutional guidelines for the care and use of animals, and all experimental procedures involving animals were approved by the Animal Experimental Committee of South China Agricultural University (SYXK2014-0136).

### Animals and Milk Sample Collection

All Large-White × Landrace pregnant sows were housed under the same conditions in a commercial farm in Guangdong Province, China. Samples were collected from April to May 2017. A total of 30 candidate healthy sows with similar expected delivery dates were selected and intramuscularly injected with cloprostenol (0.2 mg per sow) at 9:00 AM on day 113 of gestation to ensure synchronous delivery. Candidate sows were excluded if the difference in delivery time was more than 3 h. In total, 20 synchronously delivering adult pregnant multiparous sows (parity = 3∼5) were selected for this study. Sows received the same diet during gestating and lactating periods. The diet was based on corn and soybean-meal and designed to meet or exceed the energy requirement of NRC (2012), as shown in Supplementary Table S1. Sows were given *ad libitum* access to water and feed. None of the sows in the study required antibiotics during the sampling period.

Milk samples were collected from each sow on days 0 (the day of parturition), 1, 3, 5, 7, 10, 14, and 21 (*n* = 160). The milk samples from day 0 were collected before the piglets suckled their mothers’ colostrum, while the other milk samples were collected between 9:00 and 11:00 AM on the sampling day. Sterile gloves were worn during milk collection, and the nipple and surrounding area of the sow were cleaned with soap and sterile water and then cotton soaked with 75% ethyl alcohol to minimize the contamination by skin bacteria. After the first few drops (approximately 1 mL) were discarded, milk samples (approximately 15 mL) were collected manually in a sterile tube. The milk samples were immediately placed in liquid nitrogen and stored at -80°C for later analysis.

### DNA Isolation and MiSeq Sequencing

A total of 2 mL of milk sample was thawed on ice and centrifuged at 15,000 × *g* for 10 min to separate fat and cells from whey. The pellet was resuspended in 1.4 mL of ASL buffer (QIAGEN, United States). Zirconium glass beads (400 mg; diameter, 0.1 mm) (BioSpec Products, Bartlesville, OK, United States) were added to the suspension, which was then vortexed vigorously twice using a FastPrep-24 Instrument (MP Biomedicals, United States) at a speed of 6.0 m/s for 90 s. The mixture was then incubated at 95°C for 5 min (Boix-Amoros et al., 2016). The total DNA was then isolated using a QIAamp^®^ DNA Stool Kit (QIAGEN, United States) according to the manufacturer’s protocol. The DNA was stored at -20°C until further use.

To analyze the phylogenetic composition of the bacterial community, we amplified the V4 hypervariable region of the 16S rRNA gene with universal bacterial primers (F515, 5′-GTGYCAGCMGCCGCGGTAA-3′, and R806, 5′-GGACTACNVGGGTWTCTAAT-3′) (Bokulich et al., 2012; Choy et al., 2014), where the poly-N sequence (italicized) contained an 8-nt barcode unique to each sample and a 2-nt linker sequence (bold). PCRs were carried out in triplicate using a 25 μl reaction containing 11 μl of PCR-grade water, 10 μl of 5′PRIME HotMasterMix, 3 μl of DNA template (or nuclease-free water as a negative control), and 0.5 μl of each primer (initial concentration 10 μM). PCR amplification was carried out at 94°C for 3 min; followed by 35 cycles of 94°C for 45 s, 50°C for 60 s, and 72°C for 90 s; and a final extension for 10 min at 72°C. After amplification, a Qubit^®^ 2.0 Fluorometer (Invitrogen) was used to measure the DNA concentration. The PCR products were pooled at equimolar concentrations and purified using a QIAquick PCR purification kit (QIAGEN). Purified PCR amplicon samples were paired-end (2 × 250 bp) sequenced on an Illumina MiSeq platform at the Honor Technology Company of Beijing.

### Sequence and Construction of a Co-occurrence Network

To obtain results of greater accuracy and reliability in the subsequent bioinformatic analysis, we demultiplexed and quality-filtered the raw reads using the Quantitative Insights Into Microbial Ecology (QIIME) program (v 1.9.1). Reads were trimmed and removed based on quality scores <25 and lengths >225 bp, respectively (Mao et al., 2015). After low-quality sequences were removed, the retained sequences were processed and analyzed using QIIME (v1.9.1). Chimeras and error sequences in the optimized data were removed using QIIME software (v1.9.1) by clustering the data into operational taxonomic units (OTUs) for species classification with 97% similarity (Edgar, 2010). A genus-level phylogenetic tree was constructed using the QIIME (v1.9.1) (Caporaso et al., 2010) built-in scripts and was imaged by R (v3.0.3) software. The observed OTUs, Shannon index, and Chao index were calculated to assess alpha diversity using phyloseq, and rarefaction curves were drawn using R (v3.0.3) software (Hu et al., 2016). Bray–Curtis and weighted and unweighted UniFrac principal coordinate analysis (PCoA) based on OTUs was performed by QIIME (v1.9.1) software.

### Construction of a Co-occurrence Network

To understand the interrelationships of predominant genera in sow milk samples, co-occurrence patterns of the predominant milk genera were constructed in the network interface by Spearman’s rank correlations based on bacterial abundance. A valid co-occurrence event was based on strong (Spearman’s *r*_s_ < -0.7 or *r*_s_ > 0.7) and significant (*p* < 0.01) correlations between predominant genera. Nodes in the network represented the predominant genera, and edges indicated relations between genera. The size of each node is proportional to its degree (the number of connections) in our dataset.

### Milk Composition and Spearman Rank-Order Correlation Analysis

We analyzed milk samples by spectrophotometry using MilkoScan FT 6000 (FOSS) to elucidate their fat, protein, and lactose composition (% w/w). Heat maps of Spearman rank-order correlation coefficients were constructed using the vegan and gplots packages in R (v3.0.3) software. Correlations were considered significant if *p* ≤ 0.05 when assessing multiple comparisons.

### Predicted Molecular Functions Based on 16S rRNA Data Using PICRUSt

We used Phylogenetic Investigation of Communities by Reconstruction of Unobserved States (PICRUSt) (Langille et al., 2013) to predict metagenome function using 16S rRNA marker gene sequences and referring to published complete genome sequences. OTUs were picked by a closed reference approach against the Greengenes 13_8 reference database using the pick_closed_reference_otus.py script bundle with QIIME (Campbell et al., 2010). An OTU table was then normalized based on the copy numbers of 16S rRNA genes, and metagenomes were predicted from the Kyoto Encyclopedia of Genes and Genomes (KEGG) catalog. Both R (v 3.0.3) and STAMP (v 2.0.9) were used for statistical analyses of the functional profiles. A standard *p*-value <0.05 was considered to indicate significance in all other analyses.

### Statistical Analysis

Group differences in the alpha diversity (Chao1 and Shannon index and observed OTUs) and milk composition of sow milk were calculated by one-way analysis of variance (ANOVA) using the R stats package. The statistical significance of the spatial structure of PCoA plots was calculated using an unweighted distance-based analysis of molecular variance (AMOVA). Metastats analyses were performed to compare the relative abundance of phyla and genera in milk samples at different times. All *p-*values (corrected) were calculated at 95% confidence intervals, and differences were considered significant when *p* < 0.05.

### Accession Number

The sequence information in this paper has been deposited in the European Nucleotide Archive database under accession number PRJEB24125.

## Results

### Overall Milk Microbiota Structure in the Sow

In this study, milk samples (*n* = 160) were collected from 20 sows at eight time points (0, 1, 3, 5, 7, 10, 14, and 21 days) throughout lactation to investigate changes in microbial communities. Samples that failed quality control were excluded for taxonomic classification, resulting in the analysis of 130 milk samples (12 from day 0, 13 from day 1, 15 from day 3, 18 from day 5, 19 from day 7, 20 from day 10, 18 from day 14, and 15 from day 21). A total of 1,244,965 high-quality sequences were generated from milk samples after quality control, with an average of 62,853 (range 21,905–84,857) high-quality sequences per sample. Rarefaction curves of 130 samples at the minimum cut-off of 97% sequence identity nearly plateaued, which demonstrated that the sampling depth was sufficient to characterize the microbiota in milk samples (Supplementary Figure S1).

**FIGURE 1:**
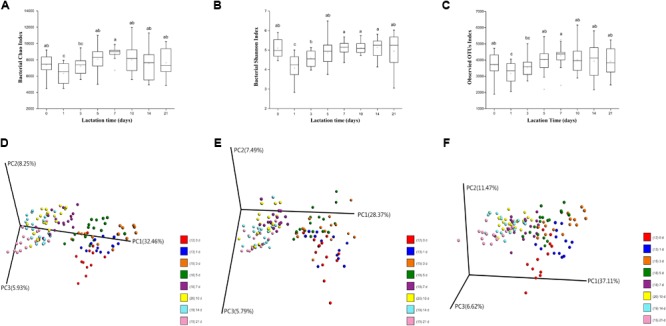
Changes in milk microbial diversity throughout lactation. **(A)** Bacterial alpha diversity determined by the Chao index. **(B)** Bacterial alpha diversity determined by the Shannon index. **(C)** Bacterial alpha diversity determined by the observed OTUs index. **(D)** PCoA based on Bray-Curtis. **(E)** PCoA based on unweighted Unifrac distances. **(F)** PCoA based on weighted Unifrac distances.

The Chao 1, Shannon and observed OTUs indices were calculated to determine the richness and diversity of the milk microbiota throughout the duration of lactation. In our study, lactation time had significant effects on the Chao 1, Shannon and observed OTUs indices (*p* < 0.01, *p* < 0.01, and *p* < 0.05, respectively), mainly attributable to changes in volatility during the first 5 days. Over time, there was a significant increase in the Chao 1, Shannon and observed OTUs indices from 1 to 5 days, with no further changes afterward (**Figures 1A–C**). In addition, the alpha diversity of the milk microbiota on day 0 was significantly higher than that on days 1 and 3. Between-group diversity was assessed using the unweighted and weighted UniFrac and Bray–Curtis distance metrics and visualized using PCoA plots. The PCoA plots show that the bacterial community profiles clustered more closely to each other in transitional and mature milk samples (from 7 to 21 days) than in colostrum samples (from 0 to 5 days) (**Figures 1D–F**). AMOVA based on unweighted distance was used to evaluate the statistical significance of the spatial separation as a function of lactation time in PCoA plots. Bacterial diversity in milk samples differed significantly with lactation time (*p* < 0.01), with the exception of similarities between days 1 and 3 (*p* = 0.266), days 7 and 10 (*p* = 0.313), days 7 and 14 (*p* = 0.804), and days 10 and 14 (*p* = 0.196) (Supplementary Table S2).

**FIGURE 2:**
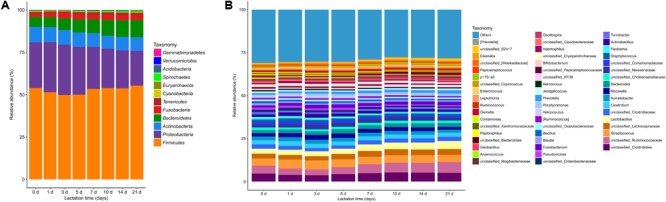
Bacterial taxonomic composition in sow milk samples (*n* = 130) throughout lactation as inferred by polymerase chain reaction amplification and pyrosequencing of 16S rRNA. **(A)** Relative abundances of the bacterial composition at the phylum level in the milk samples. **(B)** Relative abundances of the predominant bacterial composition at the genus level (≥0.5% of total sequences) in the milk samples.

Twelve microbial phyla were identified in the sow milk microbiota of all samples based on 97% 16S rRNA gene sequence identity (**Figure 2A** and Supplementary Table S3). *Firmicutes* (mean relative abundance: 52.7%) and *Proteobacteria* (25.4%) were the most abundant bacterial phyla in the sow milk samples (Supplementary Table S3). In addition to *Proteobacteria* and *Firmicutes, Bacteroidetes* (8.2%), *Actinobacteria* (8.3%), *Fusobacteria* (3.0%) and *Tenericutes* (1.1%), whose mean relative abundances accounted for greater than 1% of the total sequences, were regarded as predominant bacterial phyla. These predominant bacterial phyla accounted for 98.7% of the total sequences in the milk samples. The remaining bacterial phyla (*Acidobacteria, Cyanobacteria, Gemmatimonadetes, Spirochaetes, Verrucomicrobia*) and *Euryarchaeota*, with sequence frequencies accounting for <1% of the total sequences, were considered low abundance.

At the genera level, 212 taxa were observed in the milk microbial communities, but 36.8% of all sequences were not identified at the genera level. The 51 most predominant bacterial taxa, which were defined as having a relative abundance of more than 0.5% of total sequences, are presented for clarity and visualization purposes (**Figure 2B** and Supplementary Table S4). These 51 predominant bacterial taxa accounted for over 71% of the total sequences in sow milk. Unclassified *Ruminococcaceae* (mean relative abundance: 4.9%), which belongs to the phylum *Firmicutes*, was the most predominant taxon in milk bacterial communities (**Figure 2B** and Supplementary Table S4). *Streptococcus* (4.6%), *Lactobacillus* (3.5%), and unclassified genera derived from *Clostridiales* (4.6%) and *Lachnospiraceae* (3.1%) were the other predominant genera of *Firmicutes* (**Figure 2B** and Supplementary Table S4). Other predominant genera included *Acinetobacter* (2.1%), *Moraxella* (2.3%) and unclassified Neisseria (2.1%), which belong to the phylum *Proteobacteria*. *Bacteroides* (2.2%), *Porphyromonas* (1.1%), and *Prevotella* (1.4%) were the predominant genera of *Bacteroidetes*. *Corynebacterium* and unclassified *Micrococcaceae* were the predominant genera of *Actinobacteria* and constituted 3.1% and 1.1% of total genera (**Figure 2B** and Supplementary Table S4), respectively. *Methanobrevibacter* (0.21%) belongs to the phylum *Euryarchaeota* and was the only archaeal taxon in our samples. At the species level, a total of 50 species were detected in our samples, accounting for only 10.3% of total sequences (Supplementary Figure S4A). *Lactobacillus reuteri* (0.76%) was the most dominant species in milk bacterial communities.

### Milk Microbial Taxonomic Composition Changes With Lactation Time

**FIGURE 3:**
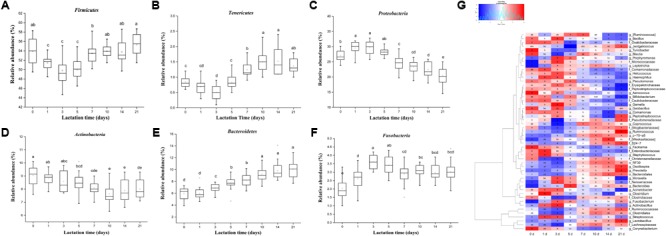
Shifts in the predominant phyla (>1% of the total sequences) and genera (≥0.5% of the total sequences) in sow milk throughout lactation (*n* = 130). **(A)** Firmicutes, **(B)** Tenericutes, **(C)** Actinobacteria, **(D)** Bacteroidetes, **(E)** Proteobacteria, and **(F)** Fusobacteria. **(G)** Changes in the relative abundances of predominant genera throughout lactation. Spot colors in the panel represent the relative abundances of predominant genera. Different letters in boxes denote significant differences between groups tested by paired sample Wilcoxon signed-rank test and adjusted by FDR.

In our study, lactation time significantly affected the relative abundance of six predominant phyla (**Figure 3**, *p* < 0.01). The relative abundance of *Firmicutes* significantly decreased from day 0 to day 5 and then generally increased with lactation time (**Figure 3A**). However, the relative abundances of *Proteobacteria* and *Fusobacteria* significantly increased from day 0 to day 5 (from 26.99 to 28.24% and from 2.03 to 3.40%, respectively) and then decreased with lactation time (from 28.24 to 20.54% and from 3.40 to 3.01%, respectively) (**Figures 3C,F**). The proportion of *Actinobacteria* significantly decreased with lactation time (from 9.03 to 8.00%) (**Figure 3D**). The relative abundance of *Bacteroidetes* significantly increased from 5.95 to 10.07% with lactation time (**Figure 3E**). In addition, the proportion of *Tenericutes* significantly increased from day 1 to day 21 (from 0.69 to 1.37%) (**Figure 3B**). The relative abundance of *Euryarchaeota* significantly decreased from 0.28 to 0.09% during the first 3 days and then generally increased from day 3 to day 21 (from 0.09 to 0.41%) (Supplementary Figure S2).

Very small but significant lactation time effects on the relative abundance of predominant genera were observed. In our study, 49 of 51 predominant taxa shifted significantly with lactation time (**Figure 3G**, *p* < 0.05). The proportions of five predominant taxa, including *Prevotella* (from 0.53 to 1.47%), unclassified *S24-7* (from 0.41 to 1.11%), *Lactobacillus* (from 2.84 to 4.68%), *Ruminococcus* (from 0.58 to 0.93%), and *p-75-a5* (from 0.53 to 0.84%), significantly increased with lactation time. A significant increase in the relative abundances of *Prevotella* and unclassified *S24-7*, belonging to the phylum *Bacteroidetes*, led to a significant increase in the proportion of the phylum *Bacteroidetes* with lactation time. However, the proportions of seven genera, specifically *Staphylococcus* (from 1.53 to 1.21%), *Facklamia* (from 1.47 to 1.04%), unclassified *Peptostreptococcaceae* (from 0.83 to 0.58%), unclassified *Erysipelotrichaceae* (from 0.82 to 0.56%), unclassified *Caulobacteraceae* (from 0.78 to 0.49%), *Comamonas* (from 0.61 to 0.43%), and *Acinetobacter* (from 2.16 to 1.79%), significantly decreased with sow lactation time. *Bifidobacterium* and *Corynebacterium* were the predominant genera of the phylum *Actinobacteria* whose relative abundances significantly decreased (from 0.83 to 0.46% and 3.66 to 3.13%, respectively), which resulted in a general decrease in the proportion of the phylum *Actinobacteria* with lactation time. The relative abundances of *Oscillospira* and unclassified genera from *Ruminococcaceae* (family), *Clostridiales* (order), *Lachnospiraceae* (family), and *Clostridiaceae* (family), which were the most predominant taxa belonging to *Firmicutes*, significantly decreased during the first 5 days and subsequently increased until day 21, significantly decreasing the relative abundance of *Firmicutes* during the period from 0 to 5 days and then increasing its proportion with lactation time. Furthermore, the proportions of the genera *Moraxella, Actinobacillus, Acinetobacter*, and unclassified *Neisseriaceae*, which were the most predominant genera belonging to *Proteobacteria*, significantly increased during the first 5 days and then decreased with lactation time, resulting in significant increases in the relative abundance of *Proteobacteria* from 0 to 5 days and gradually decreases from then until day 21. The relative abundance of *Methanobrevibacter*, which was the only archaeal taxon belonging to *Euryarchaeota*, significant decreased during the period from 0 to 3 days and then gradually increased with lactation time (Supplementary Figure S3). In addition, we further analyzed changes in abundance at the species level throughout lactation. In our study, 40 of 50 species significantly shifted across the duration of lactation (Supplementary Figure S4A). The relative abundances of *Lactobacillus reuteri, Lactobacillus mucosae*, and *Akkermansia muciniphila* significantly increased with lactation time (from 0.62 to 0.94%, from 0.14 to 0.22%, and from 0.066 to 0.16%, respectively), while *Staphylococcus epidermidis* generally decreased (from 0.20 to 0.097%) (Supplementary Figures S4B–E, *p* < 0.05). The relative abundances of most other species showed significant changes in the colostrum and were generally stable during the mid and final lactation stages.

Although community membership greatly varied, the core bacterial community that was shared in all milk samples may exert important functions. All of our samples contained 60 core bacterial taxa at the genus level, 19 unclassified taxa at the family level and 5 unclassified taxa at the order level (Supplementary Figure S5). The core microbiome in milk samples accounted for 80.6% of the total sequences. Forty-three bacterial taxa in the core microbiome were from *Firmicutes*, 6 from *Bacteroidetes*, 23 from *Proteobacteria*, 7 from *Actinobacteria*, 3 from *Fusobacteria*, 1 from *Cyanobacteria*, and 1 from *Tenericutes*. All of the predominant genera were present in the core bacterial community.

### Co-occurrence Networks of Milk Bacteria

**FIGURE 4:**
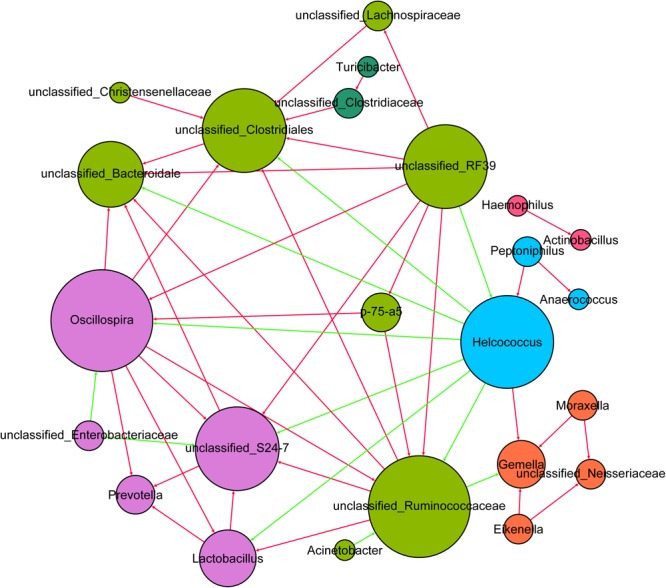
The network of co-occurring predominant genera within the milk samples (*n* = 130). The nodes represent the predominant genera, and the size of each node is proportional to the degree (the number of connections). The edges represent strong and significantly positive (red) or negative (green) correlations between predominant genera. The nodes are colored based on module structure.

To explore milk microbe interactions, we performed a milk bacterial community network analysis based on strong and significant correlations (Spearman’s *r*_s_ < -0.7 or *r*_s_ > 0.7, *p* < 0.01) at the genus level (**Figure 4**). To reduce the complexity of the network and avoid the potential impact of inter-subject variation on the network analysis, we considered only the predominant genera of the milk samples for network analysis. In this microbiome network, 24 predominant genera were identified to have significant positive or negative correlations with other genera. The milk microbe network consisted of 24 nodes (predominant genera) and 94 edges (relations) with an average degree (the mean number of connections per node) of 7.83. Six clusters (modules) were identified in the bacterial co-occurrence network in sow milk with a high degree of confidence. In this network, the proportion of *Helcococcus* had negative correlations with that of *Oscillospira, Lactobacillus*, and unclassified *Ruminococcaceae, Bacteroidales, Clostridiales, S24-7* and *RF39*. However, the proportion of *Helcococcus* had positive correlations with that of *Peptoniphilus* and *Gemella*. In our study, the *Oscillospira* relative abundance had positive correlations with that of *Prevotella, Lactobacillus*, and unclassified *Ruminococcaceae*. In addition, unclassified *Ruminococcaceae* levels were positively correlated with those of *Oscillospira, Lactobacillus*, unclassified *Clostridiales* and unclassified *Bacteroidales*. The *Lactobacillus* relative abundance also showed positive correlations with that of *Prevotella*, unclassified *Ruminococcaceae* and *Oscillospira* (**Figure 4**).

### Predicted Molecular Functions of Milk Microbiota

**FIGURE 5:**
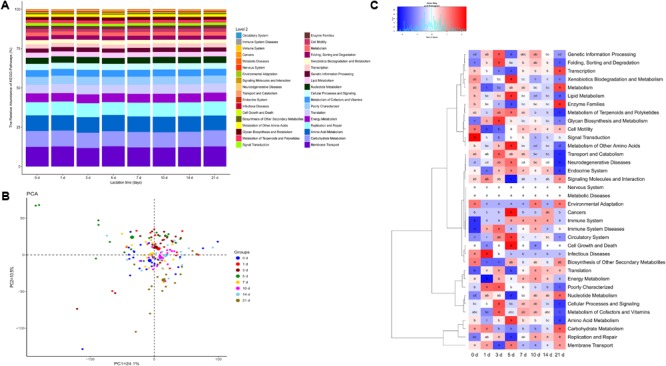
Metagenomic functional predictions for milk samples (*n* = 130). **(A)** Variations in KEGG metabolic pathways in functional bacterial communities throughout sow lactation. **(B)** PCoA of microbial functional diversity across all milk samples using the relative abundances of functional pathways. **(C)** Comparisons of the gene pathways of the bacterial microbiota throughout sow lactation.

To understand the development of the molecular functions of the milk microbial community with lactation time, we used a PICRUSt approach to predict KEGG pathway compositions of the bacterial communities. At level 1, approximately 47.5% of the genes are affiliated with metabolism, 19.6% of genes are involved with genetic information processing, and 14.3% of genes belong to environmental information processing. At level 2, 36 KEGG pathways were identified in the milk samples (**Figure 5** and Supplementary Table S5). Of the 36 gene families, the majority of the genes were associated with membrane transport (12.4%), amino acid metabolism (9.9%), carbohydrate metabolism (9.8%), replication and repair (8.7%), translation (5.7%), and energy metabolism (5.6%) (**Figure 5A**). In addition, we further analyzed the composition of gene families at level 3. A total of 328 KEGG pathways were identified, and 27 predominant metabolic pathways had relative abundances greater than 1% in our samples (Supplementary Figure S6). These profiles revealed that most pathways at level 3 were related to transporters (6.2%).

Principal coordinate analysis based on the relative abundances of the KEGG pathways of the milk microbiota identified close clustering of pathways at 0, 1, 3, 7, 10, and 14 days, and a relative divergence of pathways at 5 and 21 days (**Figure 5B**). Next, we analyzed KEGG pathways that had been annotated at level 2. The relative abundances of 28 of the 36 gene families in the milk microbiota differed significantly with lactation time (**Figure 5C**, *p* < 0.01). The proportions of genes related to infectious diseases, signal transduction and transport and catabolism significantly decreased with lactation time (**Figure 5C**, *p* < 0.01). The proportions of genes for amino acid metabolism, lipid metabolism, metabolism of other amino acids and neurodegenerative diseases significantly increased from day 0 to day 5 and then decreased with lactation time (**Figure 5C**). In contrast, the relative abundances of genes involved in nucleotide metabolism, transcription, enzyme families and signaling molecules and interaction significantly decreased for the first 5 days and then increased with lactation time (**Figure 5C**). In addition, the relative abundances of other gene families dramatically varied with lactation time. To further characterize the effects of lactation time on the proportions of functional genes, we analyzed the variations in gene families at level 3. According to our results, all the predominant pathways were affected by lactation time (Supplementary Figure S6).

### Relationships Between Predominant Bacteria and the Components of Sow Milk

**FIGURE 6:**
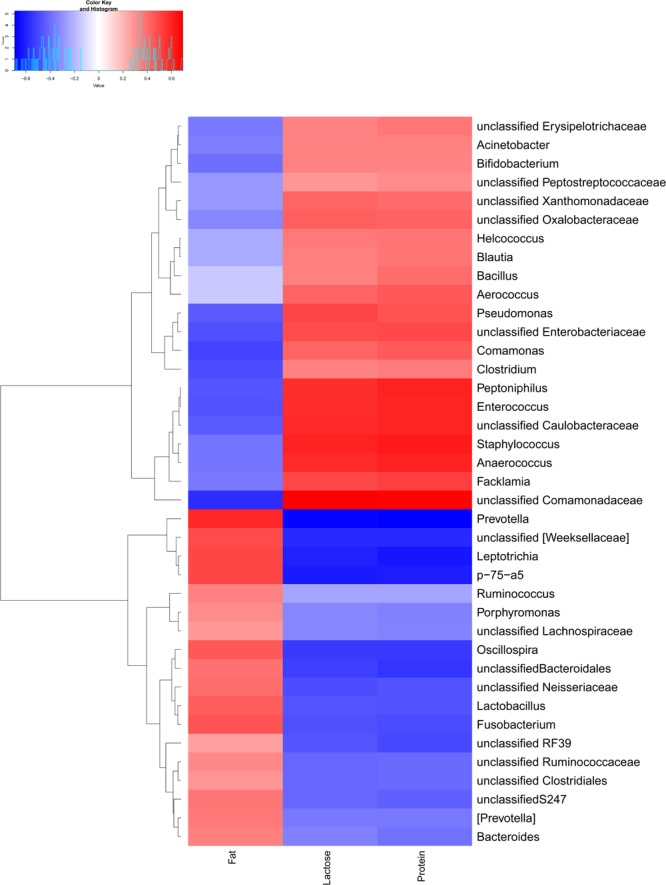
Relationships between bacterial composition and the nutritional content of sow milk (*n* = 130). The figure shows a heatmap in which samples have been clustered according to their compositional profile. Bacterial genera are color-coded according to their under- (red) or over-representation (blue) in the samples, and the proportion correlates with the protein content, fat content, and lactose content.

Spearman rank-order correlations between nutrition content (Supplementary Table S6) and the relative abundances of the predominant bacteria in milk were analyzed to determine the relationships between components (Supplementary Table S7) and the microbiota of sow milk. The abundance of 29 bacterial taxa significantly correlated with the fat content in the milk samples: 14 of these bacterial taxon abundances were significantly positively correlated (*r*_s_ ≥ 0.3 and *p* ≤ 0.05) with milk fat content, while the others were negatively correlated (*r*_s_ ≤ 0.3 and *p* ≤ 0.05) (**Figure 6**). In our study, the abundances of 20 and 21 bacterial taxa were significantly positively correlated with lactose and protein content in the milk samples, respectively, while the abundances of 17 bacterial taxa were significantly negatively correlated with both lactose and protein content (*r*_s_ ≤ 0.3 and *p* ≤ 0.05) (Supplementary Table S7). The abundances of 27 bacterial taxa were all significantly correlated with fat, lactose and protein content in milk. The bacterial taxa abundances that were positively correlated with fat content were negatively correlated with protein and lactose content in the milk samples. The relative abundances of *Prevotella* and *Leptotrichia* were the most positively correlated with fat content (*r*_s_ = 0.58, *r*_s_ = 0.50; *p* ≤ 0.01) of all the genera. Conversely, the fat content was most negatively correlated with the proportion of unclassified *Comamonadaceae* (*r*_s_ = -0.57; *p* ≤ 0.01). In addition, the relative abundances of *Staphylococcus* and unclassified *Comamonadaceae* were the most positively correlated of the genera with lactose (*r*_s_ = 0.60, *r*_s_ = 0.69; *p* ≤ 0.01) and protein content (*r*_s_ = 0.62, *r*_s_ = 0.68; *p* ≤ 0.01).

## Discussion

In this present study, alpha and beta diversity analyses indicated that lactation stage plays crucial roles in shaping the composition of the sow milk microbiota. Our findings were generally similar to those previously described for human milk (Hunt et al., 2011; Cabrera-Rubio et al., 2012; Drago et al., 2017), cow milk (Li et al., 2018) and goat milk samples (McInnis et al., 2015). However, changes in the alpha and beta diversity mainly appeared in the colostrum, with no further changes in transitional and mature milk in our study. One plausible explanation is that the proportion of nutrient volatility changes in the colostrum and is generally stable in transitional and mature milk.

To our knowledge, this is the first study to conduct a comprehensive investigation of microbial diversity and composition in sow milk. The most predominant phyla identified in sow milk were *Firmicutes* and *Proteobacteria*, and the six most predominant genera were *Ruminococcaceae, Streptococcus*, unclassified *Clostridiales, Lactobacillus, Corynebacterium*, and unclassified *Lachnospiraceae*. At the phylum level, the composition of the bacterial communities detected in the present study was similar to that described in previous studies of human milk (Urbaniak et al., 2016). Similar counter-balanced relationships between *Firmicutes* and *Proteobacteria* that were reported in cow milk (Li et al., 2018) were also observed in our samples. Although the relative abundances of these two bacteria significantly fluctuated throughout lactation, total proportions remained at a certain level (75.9–80.9%), indicating that these two microorganisms compete with each other for ecological niches. *Staphylococcus, Streptococcus*, and *Propionibacterium* are generally the predominant genera reported in human milk studies (Hunt et al., 2011; Fitzstevens et al., 2017). However, *Propionibacterium* was not a predominant genus in our samples. In addition, certain core genera, such as *Bacteroides, Ruminococcus*, and *Corynebacterium*, previously detected in human milk were also found in our samples, but more of the core genera in sow milk were not found in core human milk microbiomes. Clear differences in the composition of the microbiota in sow and human milk may be due to genetic, dietary, rearing and environmental differences among subjects (Hunt et al., 2011).

Our results showed that lactation stage had a significant effect on the relative abundances of predominant phyla and genera (except unclassified *Pseudomonadaceae* and *Micrococcaceae*), although the degree of change in the relative abundances of predominant taxa was small. Lower relative abundances at the genus level might explain these inconspicuous changes. The most predominant genus in the colostrum was *Streptococcus*, while transitional and mature milk samples were dominated by unclassified *Ruminococcaceae*. *Bifidobacterium, Staphylococcus*, and *Acinetobacter*, which are lactose-utilizing genera (Zhang et al., 2017), were more abundant in colostrum and generally decreased with the decreased lactose content in transitional and mature milk samples. *Prevotella* demonstrated the most positive correlation with fat content in our samples (*r*_s_ = 0.58; *p* ≤ 0.01) and with the intestinal microbiota of growing pigs in a previous study (Feng et al., 2015). The relative abundance of *Prevotella* increased with increasing fat content throughout lactation. *Peptoniphilus* and *Anaerococcus* metabolized peptone and amino acids to produce butyrate as a major energy source (Ezaki et al., 2001). In our study, the proportions of *Peptoniphilus* and *Anaerococcus* showed positive correlations with the protein content (*r*_s_ = 0.60, *r*_s_ = 0.60; *p* ≤ 0.01) and significantly decreased as proteins content decreased. This result was consistent with other studies (Boix-Amoros et al., 2016), supporting a correlation between nutritional components and specific components of the microbiome (Li et al., 2017; Zhang et al., 2017). Major differences in the relative abundances of the most predominant genera were mostly apparent during the first 5 days of lactation, which might be due to changes in nutritional components in the colostrum and the relative stability of nutrients in transitional and mature milk. Similar patterns of change were also observed at the species level. *Lactobacillus reuteri, Lactobacillus mucosae*, and *Akkermansia muciniphila* are potential probiotic bacteria (Urbańska and Szajewska, 2014; Pajarillo et al., 2017; Chelakkot et al., 2018). The relative abundances of these probiotics were shown to significantly increase with lactation time in sow milk and the piglet gut (Saraf et al., 2017), while the potentially pathogenic *Staphylococcus epidermidis* generally decreased in our samples (Sabaté Brescó et al., 2017). Studies investigating milk-associated probiotics might be beneficial to the development of probiotics and piglet health.

Network analysis is a powerful tool for investigating microbial interactions in complex environments. In our network analysis, *Lachnospiraceae, Ruminococcaceae*, and *Bacteroidetes S24-7* demonstrated positive correlation, potentially because all of them are short chain fatty acid (SCFA)-producing strains (Biddle et al., 2013; Ormerod et al., 2016). Growing evidence suggests that *Helcococcus* is involved in a wide spectrum of animal diseases, such as subclinical mastitis and puerperal metritis (Oikonomou et al., 2012; Locatelli et al., 2013). In our study, *Helcococcus* negatively interacted with many potential probiotics, such as *Lactobacillus, Oscillospira* (Mackie et al., 2003), *Ruminococcaceae, Bacteroidales* (Mazmanian et al., 2005), and *Bacteroidetes S24-7*, while positively correlating with other pathogens, such as *Gemella* (Borro et al., 2014) and *Peptoniphilus* (Brown et al., 2014). This suggests that milk-associated probiotics may inhibit pathogen growth. *Streptococcus, Corynebacterium, Bacteroides*, and *Acinetobacter* were the predominant bacteria in sow milk and did not play a role in maintaining interactive relationships in the network. These predominant taxa may not be functional in the network due to their high diversity in sow milk. The presence of dominant species can result in a negative relationship between species diversity and ecosystem function (Creed et al., 2009; Li et al., 2017).

PICRUSt was used to predict putative metagenomes based on 16S rRNA gene profiles and to determine the potential functions of the milk microbiota. Our predicted metagenome functional analysis showed that the most abundant functional categories included membrane transport, amino acids, carbohydrates, replication and repair, translation and energy metabolism. These results align with general metabolic functions such as amino acid, carbohydrate, and protein metabolism, which are necessary for microbe survival (Lamendella et al., 2011; Erickson et al., 2012), and are consistent with metagenomic studies of human milk (Pannaraj et al., 2017). The predominant carbohydrate in sow milk, lactose, is a potential carbon source for milk bacteria, and the presence of carbohydrate metabolism is therefore expected. The general increase in genes involved in replication and repair and translation in milk was also observed in the gut microbiota of sucking piglets (Chen et al., 2018). Although the lactation stage had a significant effect on the most microbial functions at levels 2 and 3, no obvious pattern was observed in our samples.

The origin of milk bacterial communities is complex and not fully elucidated (McGuire and McGuire, 2016). The predominant genera, *Staphylococcus* and *Streptococcus*, are typical skin bacteria, indicating that the skin might be a source of the milk microbiota (Urbaniak et al., 2016). However, many obligate anaerobic gut-associated genera such as *Bacteroides, Blautia, Ruminococcus*, and *Bifidobacterium* were also detected in our study and other studies (Jost et al., 2013), suggesting that bacterial communities in sow milk do not solely originate from the host skin or environmental sources (Hunt et al., 2011). The high percentage of anaerobic intestinal microorganisms in our milk samples indicates that parts of the milk bacterial community originated from the maternal gastrointestinal tract through the bacterial entero-mammary pathway (Rodriguez, 2014). More studies are required to confirm the source of the milk microbiota.

## Conclusion

We utilized 16S rRNA pyrosequencing to systematically characterize the sow milk microbiome in 130 samples obtained throughout lactation. Lactation stage influenced microbial composition and diversity; the microbiota during early lactation was distinct from that during the mid and late lactation stages. The microbiota of sow milk is mainly dominated by *Firmicutes* and *Proteobacteria* and the taxa *Ruminococcaceae, Streptococcus, Clostridiales, Lactobacillus, Lachnospiraceae*, and *Corynebacterium*. Furthermore, we described the biological function of the milk microbiota and identified possible correlations between nutrients and predominant bacteria. These results lay the groundwork for further studies exploring milk replacement and probiotics to reduce weaning stress for piglets during the transition to food.

## Author Contributions

XL, WC, and JdM conceived and designed the experiments. WC, JdM, NL, JG, JC, and TL contributed to sampling. WC, JdM, NL, JG, JC, and RW performed the experiments and analyzed the data. WC, JdM, JyM, and XL wrote the paper.

## Conflict of Interest Statement

The authors declare that the research was conducted in the absence of any commercial or financial relationships that could be construed as a potential conflict of interest.
