# Supplementary material for: Lactation Stage-Dependency of the Sow Milk Microbiota
Source: Front Microbiol. 2018 May 11;9:945. doi: 10.3389/fmicb.2018.00945 (PMC5958203; doi:10.3389/fmicb.2018.00945)
Supplement: Supplementary file 1 [file Data_Sheet_1.DOCX]

Supplementary Material

Lactation Stage-Dependent Dynamics of the Microbiota in Sow Milk

Wei Chen, Jiandui Mi, Ning Lv, Jinming Gao, Jian Cheng, Ruiting Wu, Jingyun Ma, Tian Lan^*^, Xindi Liao^*^

*** Correspondence:** Xindi Liao: xdliao@scau.edu.cn

# Supplementary Figures and Tables

## Supplementary Figures


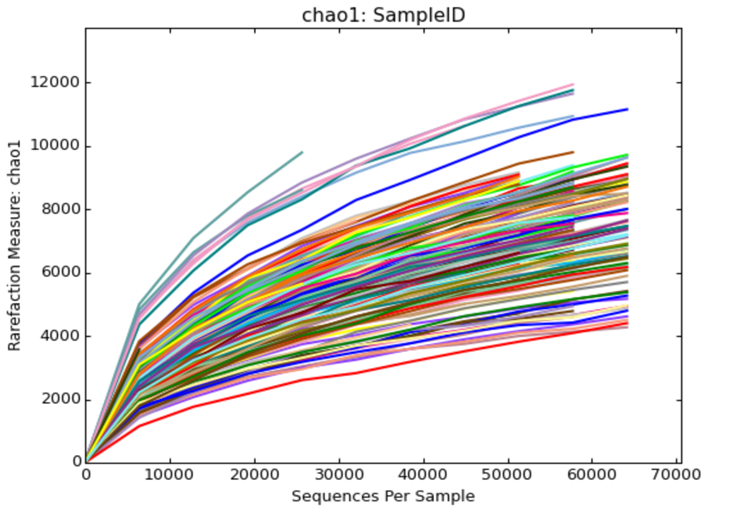


**Supplementary Figure 1.** Bacterial rarefaction curves for milk samples (n = 130) based on the Chao 1 index were used to assess the depth of coverage for each sample. Each sample is distinguished by different line colours.


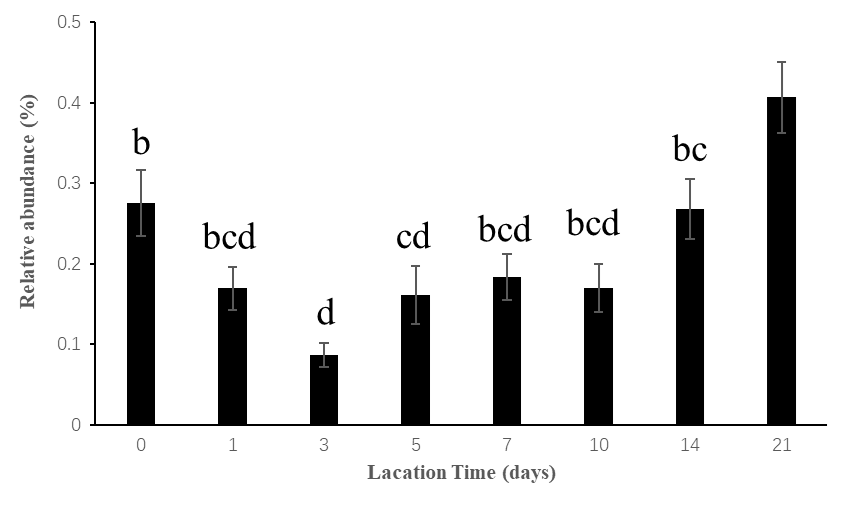


**Supplementary Figure 2.** The shifts of *Euryarchaeota* in the milk samples (n-130) during the lactation time.


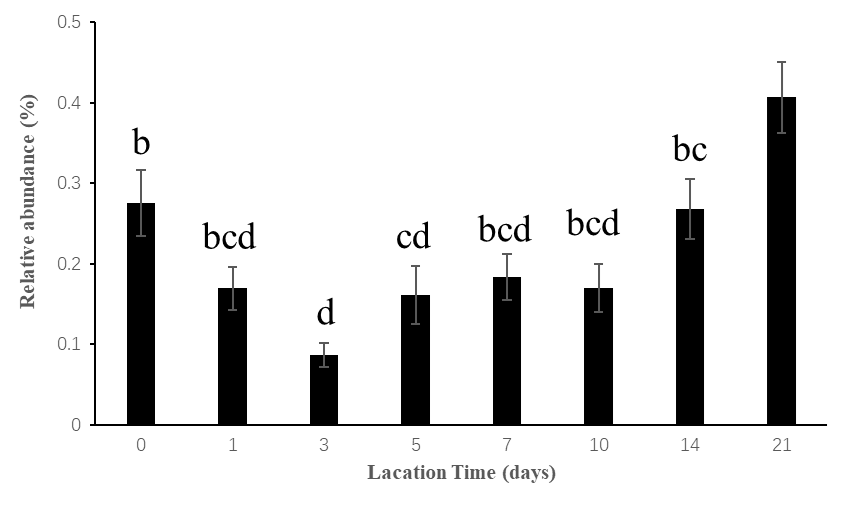


**Supplementary Figure 3.** The shifts of *Methanobrevibacter* in the milk samples (n-130) during the lactation time.


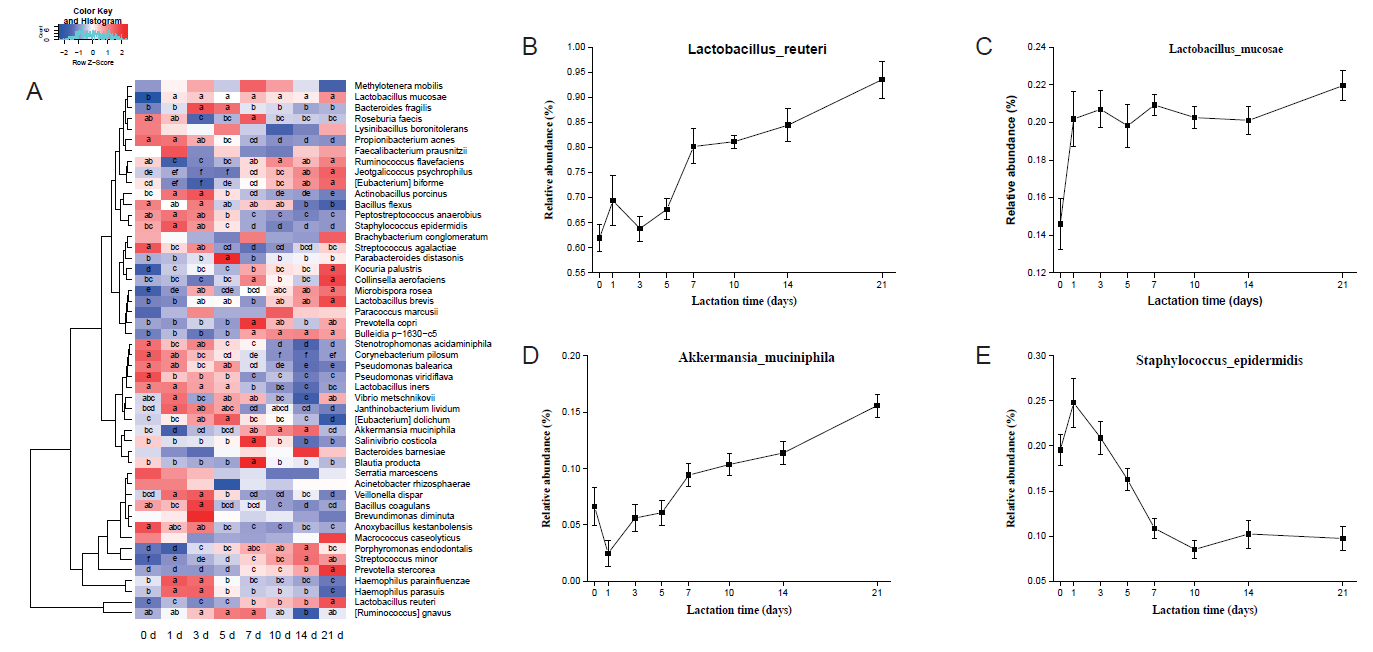


**Supplementary Figure 4.** Bacterial taxonomic composition at the species level in sow milk samples (n = 130) throughout lactation. (A) Relative abundances of bacteria at the species level. (B-E) Significant differences in the relative abundances of *Lactobacillus reuteri*, *Lactobacillus mucosae* *Akkermansia muciniphila* and *Staphylococcus* *epidermidis* throughout lactation. Spot colours in the panel indicate the relative abundances of predominant genera. Different letters in boxes denote significant differences between groups tested by a paired-sample Wilcoxon signed-rank test and adjusted by FDR.


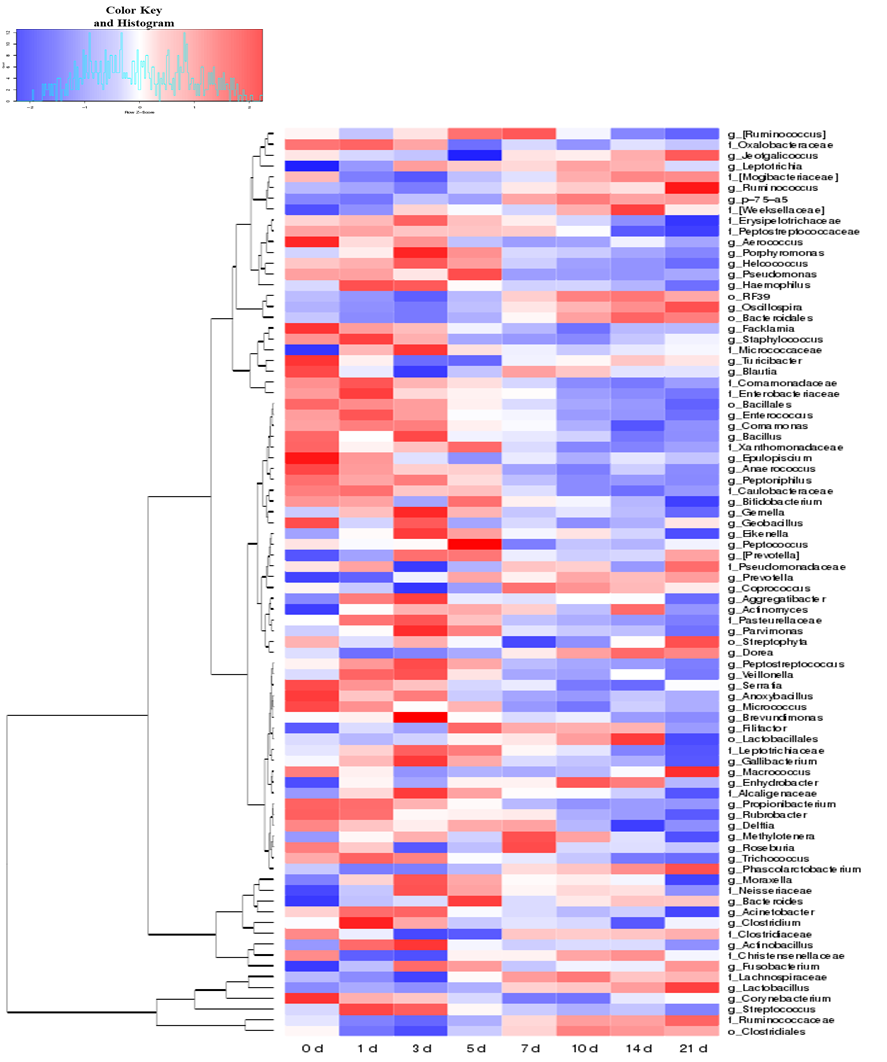


**Supplementary Figure 5.** Changes in the relative abundances of core genera throughout lactation. Spot colours in the panel indicate the relative abundances of the core genera.


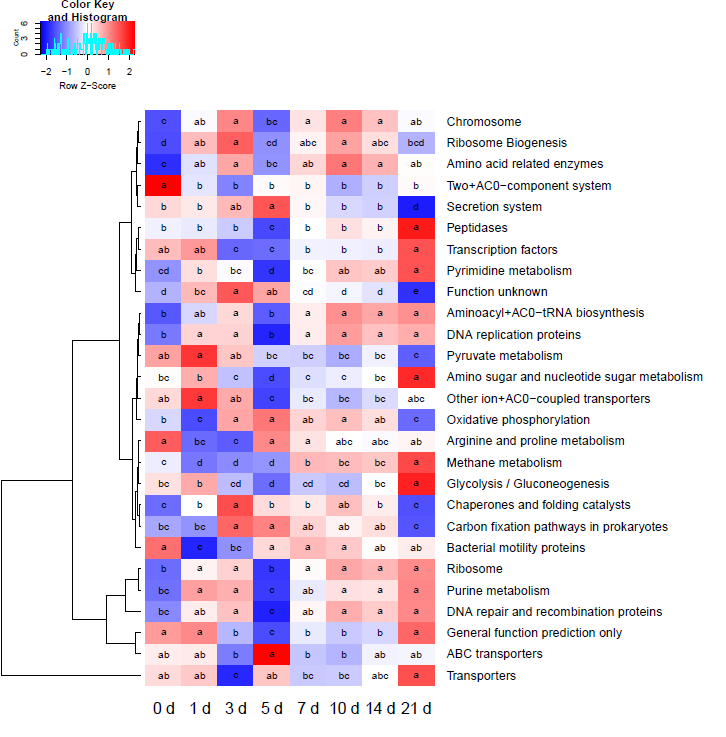


**Supplementary Figure 6.** Variations in KEGG metabolic pathways at level 3 in functional bacterial communities throughout sow lactation (n = 130). Spot colours in the panel indicate the relative abundances of predominant genera. Different letters in boxes denote significant differences between groups tested by a paired-sample Wilcoxon signed-rank test and adjusted by FDR.

## Supplementary tables

**Supplementary tables 1.** Ingredients and nutrients of the experimental diets (air-dry basis).

| Ingredients | Composition (%) | Nutrients | Contents |
| --- | --- | --- | --- |
| Corn | 57.85 | Digestible Energy (DE), MJ/kg | 13.32 |
| Soybean meal | 26.02 | Crude protein | 17.50 |
| Expanded soy | 2.03 | Calcium | 0.95 |
| Wheat bran | 4.55 | Available phosphorus | 0.48 |
| Fish meal | 1.53 | Total Lys | 1.05 |
| Oil powder | 3.00 | Total Met | 0.27 |
| NaCl | 0.40 | Total Thr | 0.65 |
| Choline chloride | 0.10 | Total trp | 0.19 |
| CaCO_3_ | 1.04 |  |  |
| CaHPO_4_ | 1.70 |  |  |
| *L*-Lys (98%) | 0.05 |  |  |
| *DL*-Met (98%) | 0.05 |  |  |
| *L*-Thr (98%) | 0.02 |  |  |
| NaHCO_3_ | 0.30 |  |  |
| Na_2_SO_4_ | 0.20 |  |  |
| Premix^1^ | 1.00 |  |  |

Premix to provide (per kg of dry matter) 12000 IU of vitamin A, 3000 IU of vitamin D3, 90 IU of vitamin E, 4.0 mg of vitamin K, 3.0 mg of vitamin B_1_, 10.0 mg of vitamin B_2_, 4.0 mg of vitamin B_6_, 40 ug of vitamin B_12_, 50.0 mg of nicotinic acid, 30.0 mg of pantothenic acid, 4.0 mg of folic acid, 0.45 mg of biotin, 30.0 mg of copper sulfate, 100.0 mg of ferrous sulfate, 100.0 mg of zinc oxide, 40.0 mg of manganese sulfate and 0.25 mg of sodium selenite.

**Supplementary tables 2.** Analysis of molecular variation hypothesis testing results in determining the difference in structure of the bacterial population of milk samples across the lactation time.

|  | 0 d | 1 d | 3 d | 5 d | 7 d | 10 d | 14 d | 21 d |
| --- | --- | --- | --- | --- | --- | --- | --- | --- |
| Milk |  |  |  |  |  |  |  |  |
| 0 d |  | <0.001* | <0.001* | 0.02* | <0.001* | <0.001* | <0.001* | 0.001* |
| 1 d | <0.001* |  | 0.266 | <0.001* | <0.001* | <0.001* | <0.001* | <0.001* |
| 3 d | <0.001* | 0.266 |  | <0.001* | <0.001* | <0.001* | <0.001* | <0.001* |
| 5 d | 0.02* | <0.001* | <0.001* |  | <0.001* | <0.001* | <0.001* | 0.141 |
| 7 d | <0.001* | <0.001* | <0.001* | <0.001* |  | 0.313 | 0.804 | <0.001* |
| 10 d | <0.001* | <0.001* | <0.001* | <0.001* | 0.313 |  | 0.196 | <0.001* |
| 14 d | <0.001* | <0.001* | <0.001* | <0.001* | 0.804 | 0.196 |  | <0.001* |
| 21 d | 0.001* | <0.001* | <0.001* | 0.141 | <0.001* | <0.001* | <0.001* |  |

**Supplementary tables 3.** Comparison of the phyla in milk samples across the lactation time of sow (the multiple comparisons results were only presented for the phyla which average relative abundance ≥1% of the total sequences). Mean in the same row with different superscripts represents a significant different (*p* < 0.05).

| Phylum | 0 d | 1 d | 3 d | 5 d | 7 d | 10 d | 14 d | 21 d | SEM | *p* value |
| --- | --- | --- | --- | --- | --- | --- | --- | --- | --- | --- |
| *Firmicutes* | 53.9ab | 51.4c | 49.8c | 50.0c | 53.5b | 53.9ab | 53.8ab | 55.4a | 0.25 | ＜0.001 |
| *Proteobacteria* | 27.0b | 29.6a | 29.7a | 28.2ab | 24.8c | 23.3cd | 22.4d | 20.5e | 0.34 | ＜0.001 |
| *Actinobacteria* | 9.0a | 8.8ab | 8.6abc | 8.5bcd | 8.1cde | 7.6e | 7.9e | 8.0de | 0.072 | ＜0.001 |
| *Bacreriodetes* | 6.0a | 6.0a | 6.9c | 7.7b | 8.2b | 9.2a | 9.8a | 10.1a | 0.16 | ＜0.001 |
| *Fusobacteria* | 2.0e | 2.6d | 3.6a | 3.4ab | 2.9cd | 3.1bc | 3.0bcd | 3.0bcd | 0.059 | ＜0.001 |
| *Tenericutes* | 0.8c | 0.7cd | 0.5d | 0.8c | 1.2b | 1.5a | 1.5a | 1.4ab | 0.041 | ＜0.001 |
| *Cyanobacteria* | 0.4 | 0.4 | 0.4 | 0.4 | 0.3 | 0.3 | 0.4 | 0.5 | 0.019 | 0.202 |
| *Spirochaetes* | 0.2 | 0.2 | 0.2 | 0.4 | 0.4 | 0.6 | 0.7 | 0.6 | 0.021 | ＜0.001 |
| *Euryarchaeota* | 0.3 | 0.2 | 0.1 | 0.2 | 0.2 | 0.2 | 0.3 | 0.4 | 0.014 | ＜0.001 |
| *Acidobacteria* | 0.2 | 0.1 | 0.2 | 0.3 | 0.2 | 0.1 | 0.1 | 0.1 | 0.011 | ＜0.001 |
| *Verrucomicrobia* | 0.1 | ND | 0.1 | 0.1 | 0.1 | 0.1 | 0.1 | 0.1 | 0.0061 | ＜0.001 |
| *Gemmatimonadetes* | ND | ND | ND | 0.0 | 0.1 | 0.1 | ND | 0.0 | 0.0025 | ＜0.001 |

ND, not detected

^*^SEM: Standard error of means.

**Supplementary tables 4.** Comparison of the predominant genera (relative abundance ≥0.5% of the total sequences) in milk samples across the lactation time of sow. Mean in the same row with different superscripts represents a significant difference (*P* < 0.05).

| Taxa | 0 d | 1 d | 3 d | 5 d | 7 d | 10 d | 14 d | 21 d | SEM | *p* value |
| --- | --- | --- | --- | --- | --- | --- | --- | --- | --- | --- |
| Actinbacteria |  |  |  |  |  |  |  |  |  |  |
| Unclassified Micrococcaceae | 1.03b | 1.15ab | 1.20a | 1.14ab | 1.11ab | 1.10ab | 1.11ab | 1.13ab | 0.13 | 0.164 |
| *Bifidobacterium* | 0.83ab | 0.85a | 0.59d | 0.86a | 0.74abc | 0.70bcd | 0.62cd | 0.46e | 0.19 | <0.001 |
| *Corynebacterium* | 3.66a | 3.37ab | 3.29b | 3.08bc | 3.40c | 3.51c | 3.2bc | 3.41bc | 0.43 | <0.001 |
| Bacteroidetes |  |  |  |  |  |  |  |  |  |  |
| Unclassified Bacteroidale | 0.68c | 0.48cd | 0.44d | 0.62cd | 0.91b | 1.18a | 1.35a | 1.32a | 0.40 | <0.001 |
| *Bacteroides* | 1.83b | 2.07ab | 2.09ab | 2.52a | 2.12ab | 2.21a b | 2.32a | 2.30a | 0.42 | <0.001 |
| *Porphyromonas* | 0.94d | 1.01cd | 1.37a | 1.30ab | 1.13bc | 1.14bc | 1.14bc | 0.99cd | 0.23 | <0.001 |
| *Prevotella* | 0.53d | 0.52d | 0.66d | 0.81c | 1.02b | 1.06b | 1.09b | 1.47a | 0.030 | <0.001 |
| *Unclassified S24-7* | 0.41d | 0.32d | 0.33d | 0.37d | 0.62c | 0.85b | 0.96b | 1.11a | 0.030 | <0.001 |
| Unclassified [Weeksellaceae] | 0.46e | 0.52de | 0.66bc | 0.62bc | 0.58cd | 0.70b | 0.79a | 0.63bc | 0.013 | <0.001 |
| Firmicutes |  |  |  |  |  |  |  |  |  |  |
| *Bacillus* | 1.16a | 0.94b | 1.24a | 0.88bc | 0.94b | 0.91bc | 0.71c | 0.70c | 0.029 | <0.001 |
| *Geobacillus* | 0.69a | 0.54bc | 0.68ab | 0.51c | 0.53bc | 0.49c | 0.49c | 0.60abc | 0.018 | 0.015 |
| *Jeotgalicoccus* | 0.89abc | 0.85bc | 0.82c | 0.70d | 0.90abc | 0.89bc | 0.93ab | 0.99a | 0.014 | <0.001 |
| *Staphylococcus* | 1.53a | 1.72a | 1.48a | 1.14b | 1.07b | 1.02b | 1.15b | 1.21b | 0.034 | <0.001 |
| *Gemella* | 0.59c | 0.67b | 0.78a | 0.68b | 0.58c | 0.61c | 0.58c | 0.51d | 0.0090 | <0.001 |
| *Facklamia* | 1.47a | 1.32b | 1.27bc | 1.14cd | 1.05de | 0.96e | 1.06de | 1.04de | 0.022 | <0.001 |
| *Lactobacillus* | 2.84d | 3.04d | 2.87d | 3.04d | 3.75c | 3.81bc | 4.05b | 4.68a | 0.063 | <0.001 |
| *Streptococcus* | 4.19b | 5.30a | 5.24a | 4.58b | 4.41b | 4.33b | 4.48b | 4.14b | 0.059 | <0.001 |
| Unclassified Clostridiales | 4.64cd | 3.85e | 3.66e | 4.32d | 4.83bc | 5.29a | 5.17ab | 5.09ab | 0.071 | <0.001 |
| Unclassified Christensenellaceae | 1.57a | 1.18b | 1.16b | 1.43a | 1.43a | 1.54a | 1.56a | 1.39a | 0.024 | <0.001 |
| Unclassified Clostridiaceae | 2.51a | 2.19b | 1.83c | 1.86c | 2.38ab | 2.36ab | 2.39ab | 2.42a b | 0.033 | <0.001 |
| *Clostridium* | 2.43bc | 2.73a | 2.55b | 2.33cd | 2.37bc | 2.35cd | 2.16d | 2.40bc | 0.025 | <0.001 |
| Unclassified Lachnospiraceae | 2.85cd | 2.69de | 2.43e | 3.08bc | 3.40a | 3.51a | 3.28ab | 3.41a | 0.045 | <0.001 |
| *Blautia* | 1.22a | 0.94bc | 0.74d | 0.89cd | 1.14ab | 1.06abc | 0.94bc | 0.95bc | 0.025 | <0.001 |
| *Coprococcus* | 0.54ab | 0.47bc | 0.34d | 0.44c | 0.63a | 0.61a | 0.58a | 0.54ab | 0.014 | <0.001 |
| *[Ruminococcus]* | 0.96ab | 0.88b | 0.96ab | 1.04a | 1.06a | 0.94ab | 0.84b | 0.83b | 0.017 | <0.001 |
| Unclassified Peptostreptococcaceae | 0.83a | 0.82a | 0.80a | 0.80a | 0.81a | 0.76a | 0.62b | 0.58b | 0.012 | <0.001 |
| *Peptostreptococcus* | 0.52c | 0.55bc | 0.60ab | 0.54bc | 0.54bc | 0.48cd | 0.44d | 0.63a | 0.0099 | <0.001 |
| Unclassified Ruminococcaceae | 4.58c | 3.67de | 3.42e | 4.09cd | 5.19b | 5.72ab | 5.71ab | 6.27a | 0.11 | <0.001 |
| *Oscillospira* | 0.78de | 0.66ef | 0.59f | 0.82d | 1.08c | 1.24b | 1.36b | 1.59a | 0.033 | <0.001 |
| *Ruminococcus* | 0.58c | 0.57c | 0.50c | 0.61bc | 0.69b | 0.72b | 0.71b | 0.93a | 0.017 | <0.001 |
| Unclassified Mogibacteriaceae | 0.74a | 0.58b | 0.55b | 0.62b | 0.64b | 0.75a | 0.76a | 0.78a | 0.013 | <0.001 |
| *Helcococcus* | 0.94b | 0.99b | 1.19a | 1.03b | 0.67c | 0.56cd | 0.52d | 0.44d | 0.027 | <0.001 |
| *Turicibacter* | 1.36a | 1.12b | 0.92c | 0.92c | 1.07b | 1.11b | 1.17b | 1.13b | 0.021 | <0.001 |
| Unclassified Erysipelotrichaceae | 0.82ab | 0.83ab | 0.92a | 0.86ab | 0.79abc | 0.73bc | 0.67cd | 0.56d | 0.014 | <0.001 |
| *p-75-a5* | 0.53b | 0.51b | 0.62b | 0.57b | 0.82a | 0.89a | 0.85a | 0.84a | 0.016 | <0.001 |
| Fusobacteria |  |  |  |  |  |  |  |  |  |  |
| *Fusobacterium* | 1.22d | 1.54c | 2.07a | 1.96ab | 1.57c | 1.67c | 1.69bc | 1.95ab | 0.038 | <0.001 |
| *Leptotrichia* | 0.56c | 0.76b | 1.15a | 1.07a | 1.04a | 1.16a | 1.11a | 0.88b | 0.025 | <0.001 |
| Proteobacteria |  |  |  |  |  |  |  |  |  |  |
| Unclassified Caulobacteraceae | 0.78a | 0.80a | 0.69ab | 0.71a | 0.58bc | 0.50c | 0.45c | 0.49c | 0.018 | <0.001 |
| Unclassified Comamonadaceae | 1.56b | 1.74a | 1.45bc | 1.35c | 1.13d | 0.92e | 0.90e | 0.99de | 0.027 | <0.001 |
| *Comamonas* | 0.61ab | 0.62a | 0.61a | 0.52bc | 0.51c | 0.45cd | 0.36d | 0.43cd | 0.010 | <0.001 |
| Unclassified Oxalobacteraceae | 1.01a | 1.01a | 0.98a | 0.82b | 0.89ab | 0.87ab | 0.90ab | 0.88ab | 0.017 | 0.047 |
| Unclassified Neisseriaceae | 1.56e | 1.91cd | 2.55a | 2.32b | 2.11bc | 2.17b | 2.17b | 1.72de | 0.037 | <0.001 |
| Unclassified Enterobacteriaceae | 1.36b | 1.59a | 1.22bc | 1.14c | 1.12c | 0.87d | 0.83d | 0.77d | 0.028 | <0.001 |
| *Actinobacillus* | 1.46bc | 2.06a | 2.19a | 1.67b | 1.53bc | 1.55bc | 1.55bc | 1.35c | 0.033 | <0.001 |
| *Haemophilus* | 0.78b | 1.29a | 1.26a | 0.89b | 0.76b | 0.77b | 0.71b | 0.51c | 0.029 | <0.001 |
| *Acinetobacter* | 2.16ab | 2.31a | 2.31a | 2.08bc | 2.04bc | 1.98c | 2.02bc | 1.79d | 0.023 | <0.001 |
| *Moraxella* | 1.88d | 2.42bc | 2.83a | 2.57ab | 2.29bc | 2.36bc | 2.26c | 1.61e | 0.043 | <0.001 |
| Unclassified Pseudomonadaceae | 0.51ab | 0.52a | 0.45b | 0.50ab | 0.51ab | 0.53a | 0.49ab | 0.55a | 0.0075 | 1.000 |
| *Pseudomonas* | 1.33a | 1.25a | 1.09b | 1.38a | 0.75c | 0.76c | 0.67c | 0.73c | 0.071 | <0.001 |
| Tenericutes |  |  |  |  |  |  |  |  |  |  |
| Unclassified RF39 | 0.84c | 0.69cd | 0.51d | 0.83c | 1.24b | 1.50a | 1.52a | 1.37ab | 0.041 | <0.001 |

^*^SEM: Standard error of means.

**Supplementary tables 5.** Predicted functions of the milk bacterial microbiota of sow across the lactation time.

| Functions | 0 d | 1 d | 3 d | 5 d | 7 d | 10 d | 14 d | 21 d | SEM | P value |
| --- | --- | --- | --- | --- | --- | --- | --- | --- | --- | --- |
| **Metabolism** |  |  |  |  |  |  |  |  |  |  |
| Amino Acid Metabolism | 9.93b | 9.67c | 9.91b | 10.31a | 9.93b | 9.89b | 9.83bc | 9.44d | 0.030 | <0.001 |
| Carbohydrate Metabolism | 10.05a | 10.13a | 9.65b | 9.56b | 9.73b | 9.63b | 9.77b | 10.22a | 0.0034 | <0.001 |
| Energy Metabolism | 5.62a | 5.42a | 5.66a | 5.59a | 5.66a | 5.68a | 5.64a | 5.55a | 0.0021 | 0.076 |
| Xenobiotics Biodegradation and Metabolism | 2.63ab | 2.36c | 2.44bc | 2.82a | 2.42bc | 2.29c | 2.39c | 2.23c | 0.030 | <0.001 |
| Metabolism of Cofactors and Vitamins | 4.29abc | 4.24bc | 4.39a | 4.29abc | 4.35ab | 4.34ab | 4.31abc | 4.21c | 0.015 | 0.05 |
| Nucleotide Metabolism | 3.88cd | 4.07ab | 4.03ab | 3.78d | 4.01bc | 4.07ab | 4.06ab | 4.18a | 0.021 | <0.001 |
| Lipid Metabolism | 3.22b | 3.14bc | 3.20bc | 3.34a | 3.16bc | 3.10cd | 3.13bc | 3.03d | 0.013 | <0.001 |
| Enzyme Families | 2.01b | 1.97bc | 1.92c | 1.85d | 1.96bc | 1.98bc | 1.96bc | 2.11a | 0.0097 | <0.001 |
| Glycan Biosynthesis and Metabolism | 1.88c | 2.14b | 2.28a | 2.07b | 2.02bc | 2.06b | 2.01bc | 1.89c | 0.018 | <0.001 |
| Metabolism of Terpenoids and Polyketides | 1.88ab | 1.85b | 1.91ab | 1.92a | 1.88ab | 1.85b | 1.85b | 1.79c | 0.0076 | <0.001 |
| Metabolism of Other Amino Acids | 1.68b | 1.65bc | 1.70b | 1.78a | 1.66bc | 1.63cd | 1.65bc | 1.59d | 0.0080 | <0.001 |
| Biosynthesis of Other Secondary Metabolites | 0.74a | 0.63c | 0.63c | 0.72ab | 0.68b | 0.70ab | 0.68b | 0.73ab | 0.0062 | <0.001 |
| **Environmental Information Processing** |  |  |  |  |  |  |  |  |  |  |
| Membrane Transport | 12.54a | 12.77a | 11.80a | 12.91a | 12.19a | 12.13a | 12.35a | 12.78a | 0.10 | 0.078 |
| Signal Transduction | 1.88a | 1.70b | 1.64b | 1.71b | 1.72b | 1.66b | 1.68b | 1.70b | 0.013 | 0.01 |
| Signaling Molecules and Interaction | 0.20ab | 0.21ab | 0.20ab | 0.16c | 0.19ab | 0.19b | 0.19ab | 0.21a | 0.0029 | <0.001 |
| **Genetic Information Processing** |  |  |  |  |  |  |  |  |  |  |
| Replication and Repair | 8.37bc | 8.75ab | 8.87a | 8.17c | 8.73ab | 8.96a | 8.87a | 8.99a | 0.050 | <0.001 |
| Folding, Sorting and Degradation | 2.48c | 2.62b | 2.76a | 2.57bc | 2.62b | 2.65ab | 2.61b | 2.45c | 0.016 | <0.001 |
| Transcription | 2.67b | 2.61b | 2.49c | 2.45c | 2.63b | 2.64b | 2.63b | 2.79a | 0.014 | <0.001 |
| Translation | 5.37b | 5.75a | 5.89a | 5.34b | 5.73a | 5.90a | 5.84a | 5.83a | 0.040 | <0.001 |
| **Cellular Processes** |  |  |  |  |  |  |  |  |  |  |
| Transport and Catabolism | 0.28ab | 0.27ab | 0.30a | 0.28ab | 0.28ab | 0.26bc | 0.26bc | 0.23c | 0.0041 | 0.09 |
| Cell Motility | 2.25a | 1.59b | 1.66b | 1.98a | 2.11a | 2.06a | 2.03a | 2.11a | 0.037 | <0.001 |
| Cell Growth and Death | 0.51a | 0.50a | 0.51a | 0.53a | 0.51a | 0.50a | 0.51a | 0.51a | 0.0024 | 0.062 |
| **Human Diseases** |  |  |  |  |  |  |  |  |  |  |
| Infectious Diseases | 0.43a | 0.45a | 0.41b | 0.40b | 0.40b | 0.40b | 0.40b | 0.40b | 0.0031 | <0.001 |
| Neurodegenerative Diseases | 0.24d | 0.26cd | 0.31ab | 0.33a | 0.27cd | 0.29bc | 0.27cd | 0.18e | 0.0052 | <0.001 |
| Cancers | 0.10b | 0.10b | 0.10b | 0.12a | 0.10b | 0.10b | 0.11ab | 0.10b | 0.0016 | 0.041 |
| Immune System Diseases | 0.05b | 0.09a b | 0.10ab | 0.07a | 0.08a | 0.09a | 0.07a | 0.08ab | 0.0036 | 0.059 |
| Metabolic Diseases | 0.10 | 0.10 | 0.10 | 0.10 | 0.10 | 0.10 | 0.10 | 0.10 | 0.00070 | 1.00 |
| **Organismal Systems** |  |  |  |  |  |  |  |  |  |  |
| Endocrine System | 0.30 a | 0.27b | 0.31a | 0.32a | 0.31a | 0.30a | 0.31a | 0.26b | 0.0035 | <0.001 |
| Environmental Adaptation | 0.11a | 0.10a | 0.10a | 0.10a | 0.10a | 0.11 a | 0.10a | 0.11a | 0.0015 | 0.237 |
| Circulatory System | 0.00c | 0.01c | 0.05ab | 0.07a | 0.02c | 0.02 c | 0.03bc | 0.00 c | 0.0038 | <0.001 |
| Immune System | 0.08a | 0.09 a | 0.09a | 0.10a | 0.10a | 0.10a | 0.10a | 0.09a | 0.0017 | 0.222 |
| Nervous System | 0.10 | 0.10 | 0.10 | 0.10 | 0.10 | 0.10 | 0.10 | 0.10 | 0.0011 | 1.00 |
| **Unclassified** |  |  |  |  |  |  |  |  |  |  |
| Poorly Characterized | 5.14b | 5.31a | 5.32a | 5.17b | 5.17b | 5.14b | 5.12b | 5.04c | 0.012 | <0.001 |
| Cellular Processes and Signaling | 3.93bc | 3.95b | 4.09a | 3.91bc | 4.00ab | 4.01ab | 3.92bc | 3.79c | 0.017 | 0.001 |
| Genetic Information Processing | 2.68cd | 2.79ab | 2.87a | 2.64d | 2.78abc | 2.82ab | 2.75bc | 2.68cd | 0.013 | <0.001 |
| Metabolism | 2.39ab | 2.31c | 2.33bc | 2.38ab | 2.38ab | 2.36bc | 2.35bc | 2.43a | 0.0079 | 0.005 |

Means with same superscript within the same row are not significantly different at *p* < 0.05.

^*^SEM: Standard error of means.

**Supplementary tables 6.** The concentration of milk composition of sows at different time point.

|  | Day 0 | Day 1 | Day 3 | Day 5 | Day 7 | Day 10 | Day 14 | Day 21 | SEM | P value |
| --- | --- | --- | --- | --- | --- | --- | --- | --- | --- | --- |
| Fat (%) | 4.47 d | 5.14 cd | 8.95 a | 6.76 bc | 8.18 ab | 7.00 b | 7.33 ab | 7.51 ab | 0.28 | <0.001 |
| Protein (%) | 8.76 a | 5.58 b | 4.19 c | 4.10 c | 4.07 c | 4.02 c | 3.96 c | 4.12 c | 0.26 | <0.001 |
| Lactose (%) | 12.65 a | 8.30 b | 6.27 c | 6.19 c | 6.12 c | 6.08 c | 5.97 c | 6.20 c | 0.36 | <0.001 |

Means with same superscript within the same row are not significantly different at *p* < 0.05.

^*^SEM: Standard error of means.

**Supplementary tables 7.** Relationships between bacterial composition and nutritional content of sow milk.

|  | Fat | | Lactose | | Protein | |
| --- | --- | --- | --- | --- | --- | --- |
| Taxa | *r*_s_ | *p* value | *r*_s_ | *p* value | *r*_s_ | *p* value |
| *Prevotella* | 0.58 | <0.001 | -0.68 | <0.001 | -0.69 | <0.001 |
| *Leptotrichia* | 0.50 | 0.004 | -0.60 | <0.001 | -0.63 | <0.001 |
| *p-75-a5* | 0.50 | 0.004 | -0.62 | <0.001 | -0.61 | <0.001 |
| *Unclassified [Weeksellaceae]* | 0.49 | 0.004 | -0.58 | <0.001 | -0.58 | <0.001 |
| *Fusobacterium* | 0.47 | 0.005 | -0.47 | 0.002 | -0.49 | 0.001 |
| *Oscillospira* | 0.46 | 0.006 | -0.53 | <0.001 | -0.54 | <0.001 |
| *Lactobacillus* | 0.44 | 0.007 | -0.46 | 0.003 | -0.47 | 0.003 |
| *Unclassified Neisseriaceae* | 0.40 | 0.019 | -0.48 | 0.002 | -0.47 | 0.003 |
| *Unclassified Bacteroidales* | 0.39 | 0.023 | -0.52 | <0.001 | -0.55 | <0.001 |
| *Unclassified S247* | 0.38 | 0.024 | -0.42 | 0.008 | -0.43 | 0.006 |
| *[Prevotella]* | 0.36 | 0.030 | -0.37 | 0.023 | -0.36 | 0.022 |
| *Bacteroides* | 0.35 | 0.035 | -0.34 | 0.033 | -0.39 | 0.014 |
| *Unclassified Oxalobacteraceae* | -0.33 | 0.048 | 0.43 | 0.006 | 0.43 | 0.007 |
| *Acinetobacter* | -0.35 | 0.037 | 0.34 | 0.034 | 0.34 | 0.030 |
| *Facklamia* | -0.36 | 0.030 | 0.49 | 0.002 | 0.52 | <0.001 |
| *Unclassified Erysipelotrichaceae* | -0.37 | 0.029 | 0.34 | 0.033 | 0.37 | 0.020 |
| *Staphylococcus* | -0.38 | 0.025 | 0.60 | <0.001 | 0.62 | <0.001 |
| *Anaerococcus* | -0.38 | 0.024 | 0.58 | <0.001 | 0.60 | <0.001 |
| *Bifidobacterium* | -0.39 | 0.022 | 0.34 | 0.033 | 0.34 | 0.030 |
| *Unclassified Caulobacteraceae* | -0.45 | 0.006 | 0.58 | <0.001 | 0.59 | <0.001 |
| *Pseudomonas* | -0.45 | 0.006 | 0.50 | <0.001 | 0.47 | 0.003 |
| *Peptoniphilus* | -0.46 | 0.005 | 0.56 | <0.001 | 0.60 | <0.001 |
| *Enterococcus* | -0.47 | 0.005 | 0.57 | <0.001 | 0.59 | <0.001 |
| *Unclassified Enterobacteriaceae* | -0.48 | 0.005 | 0.48 | 0.002 | 0.49 | <0.001 |
| *Clostridium* | -0.48 | 0.004 | 0.33 | 0.035 | 0.36 | 0.023 |
| *Comamonas* | -0.51 | 0.004 | 0.42 | 0.008 | 0.45 | 0.004 |
| *Unclassified Comamonadaceae* | -0.57 | <0.001 | 0.69 | <0.001 | 0.68 | <0.001 |
